# The Importance of the First Year of War in Mental Disease

**Published:** 1941

**Authors:** R. E. Hemphill

**Affiliations:** Assistant Medical Officer, Bristol Mental Hospital; Formerly Physician-in-charge, Barrow Hospital, Bristol


### THE IMPORTANCE OF THE FIRST YEAR OF WAR IN MENTAL DISEASE.

BY

R. E. HEMPHILL, M.D., D.P.M.,

*Assistant Medical Officer, Bristol Mental Hospital;*
*Formerly Physician-in-charge, Barrow Hospital, Bristol.*

---

THE importance of war as a factor in the causation of mental illness has been extensively studied in the past. Most investigators have come to the same general conclusions, namely that amongst serving troops there is no marked increase in the incidence of the severer forms of mental illness but a considerable increase in the less severe disorders, such as anxiety states and hysteria.

A distinction, which has not been properly recognized, must be made between military service and actual warfare. It seems probable that men predisposed to the severer forms of mental illness might be rejected early in military service, and that the neurotic conditions occur largely amongst conscripts who are unsuited for military life and who develop them as a sort of environmental or occupational reaction against a mode of life for which they have a conscious or unconscious dislike. The genuine importance of warfare should be assessed from a study of the cases of mental disorder occurring amongst regular troops who have been subjected to battle.

The present war has introduced fresh factors and has been brought home to the civilian population, male and female alike, in a way that no previous war, involving this country, has been. Before the war, expressions such as " when the bombing starts every one will go crazy " were commonly heard. It was assumed that the mental hospitals and psychiatric clinics would be filled with fresh mental cases and voluntary patients seeking security and relief from the greatly increased responsibilities of private life, and that that section of the general public predisposed to mental illness would break down like the conscript soldiers and in at least as high a proportion. If this is not the case, and up to the present it appears not to be so, it may in some measure be because the average civilians feel that this war is personal and inescapable and that the management of the battle is as much in their own hands as the usual struggle for employment and existence.

In this paper a survey is made of the male and female patients admitted annually to the Bristol Mental Hospital for ten years, up to 31st December, 1940, as shown in the accompanying table. These figures are some guide to the amount of fresh mental illness occurring annually. All the conditions of severe aerial war on the civilian population have been experienced by Bristol since June, 1940. There have been daylight and severe night air-raids, fluctuations in prosperity and employment, housing difficulties and a great influx of refugees, occupational and apprehensive. The immediate psychogenic effect of these conditions should be reflected by the numbers of patients admitted to the Mental Hospital since the outbreak of the war.

Certain factors which, apart from the war, influence the rate of admission to the Mental Hospital must be borne in mind if fallacious deductions are to be avoided. In Bristol, as in most other cities since the last war, the annual admission rate to the Mental Hospital has shown a fairly steady rise each year. In 1930 the Mental Treatment Act enabled patients to be admitted to hospital on a voluntary or temporary basis without the usual forms of certification. As these forms of admission became better known and employed, the Mental Hospital population was increased annually by cases of early or mild mental illness, and others, it must be confessed, attracted by the semi-monastic advantages and comforts of a life free from responsibilities without the stigma of certified insanity. Although the table covers ten years, the figures for the last five years only give a fair picture of the incidence of recent mental illness in Bristol, for it was not until about 1934 that the Mental Treatment Act was well known and in full operation.

From the end of 1938 until the outbreak of the war, the Mental Hospital accommodation was increased by the opening of Barrow Hospital, constructed for the reception of about 380 mental patients of both sexes, where acute sickness and the earlier or more recoverable types of mental illness could be treated in conditions much more favourable than those prevailing in the old-fashioned mental hospitals. There is no doubt that Barrow Hospital attracted many voluntary patients who would not entertain the idea of going to Fishponds. The figures for patients admitted during 1939 are therefore rather exceptional and err on the high side when compared with 1940 or the earlier years.

Most patients, including nearly all the certified, admitted to the Mental Hospital have first been placed in Stapleton Institution for observation, and subsequently have been certified or have entered Fishponds voluntarily. A considerable number of the cases placed in Stapleton Institution for observation never reach the Mental Hospital. This number was probably high in 1940 because of the known overcrowding at Fishponds and the urgent necessity to economize beds. As Stapleton Institution generously accommodated some cases that required supervision rather than special treatment this fact explains, to a certain extent, the low admission rate for 1940.

At the outbreak of war more than 150 mild chronic, or partially recovered patients were sent home or transferred to Stapleton Institution under Section 25 of the Lunacy Act. Since then about fifty more patients have been transferred to Stapleton Institution. It was to be expected that of this 200 or so, a number would relapse or become unmanageable and return to the Mental Hospital as new certified admissions. This has been the case and these re-admissions make the actual number of patients admitted during 1940 fallaciously high.

ANNUAL ADMISSIONS, BRISTOL MENTAL HOSPITAL, 1930–41.

| Year. | MALES. Voluntary. | Certified. | Temporary. | Total. | FEMALES. Voluntary. | Certified. | Temporary. | Total. | Combined Total. | Recovery | Recovery per cent. of Admissions |
|---|---|---|---|---|---|---|---|---|---|---|---|
| 1940 | 38 | 135 | 2 | 175 | 42 | 128 | 9 | 179 | 354 | 118 | 33% |
| 1939 | 74 | 103 | 14 | 191 | 111 | 124 | 19 | 254 | 445 | 131 | 29% |
| 1938 | 72 | 104 | 4 | 180 | 144 | 106 | 24 | 274 | 454 | 147 | 32% |
| 1937 | 39 | 111 | 4 | 154 | 81 | 95 | 34 | 210 | 364 | 129 | 35% |
| 1936 | 56 | 112 | 6 | 174 | 91 | 112 | 21 | 224 | 398 | 131 | 33% |
| 1935 | 30 | 89 | 10 | 129 | 60 | 120 | 15 | 195 | 324 | 122 | 38% |
| 1934 | 20 | 111 | 6 | 137 | 44 | 146 | 16 | 206 | 343 | 106 | 31% |
| 1933 | 18 | 138 | 1 | 157 | 32 | 155 | 6 | 193 | 350 | 93 | 25% |
| 1932 | 6 | 113 | 1 | 120 | 24 | 146 | 9 | 179 | 299 | 104 | 35% |
| 1931 | 3 | 119 | 6 | 128 | 9 | 136 | 19 | 164 | 292 | 93 | 32% |

*Total annual admissions.*—The table shows that 354 patients, male and female, were admitted during the year 1939–40. This is the lowest total in five years, and is 100 lower than 1938, the year before patients were admitted to Barrow Hospital. As pointed out before, the general tendency has been for the annual admission rate to show a progressive rise. On this account the sharp reduction in 1940 is all the more remarkable. It will be seen that the number of male patients admitted in 1940 is the third highest for ten years, while that of females is the lowest in eight: the fall in the admission rate is therefore confined to female patients.

*Voluntary admissions.*—Patients are admitted voluntarily if they have sufficient insight to present themselves for treatment or supervision. They are therefore of two types; those in whom a mental illness of which they are aware is developing, and those who are unable or unwilling to bear the responsibility of life outside the hospital. This latter class is not entirely composed of malingerers, as the occurrence of occasional suicides amongst them shows. On the whole there is a tendency for weak-willed or neurotic patients to return to hospital when conditions become hard. One would expect, therefore, to find a great reunion in the hospital of many old voluntary patients and a marked increase in the new voluntary admissions during the period of aerial warfare. It is surprising that the total of male voluntary patients admitted in 1940 was the lowest for five years and slightly more than half of the 1938 figure, that the female figure for 1940 was the lowest for seven years and slightly less than one-third of the 1938 figure.

*Temporary admissions.*—The temporary certificate covers patients who at the time of certification are not capable of expressing a wish either for or against receiving treatment. Such patients may suffer from acute confusional states following epileptic fits, toxic conditions, the effect of exhaustion or accidents, severe mental or physical distress, or alcoholism. One would have expected all these conditions to have been prevalent since the outbreak of the war. The table shows that two men and nine women, the lowest numbers for seven years respectively, were admitted in 1940. Probably the temporary certificate has not been extensively employed in 1940 because of the inconvenience of obtaining two medical certificates, and a few of the patients certified in the ordinary way might have appeared in the temporary class, thereby increasing the number of temporary and decreasing the number of certified patients shown in the table for that year.

*Certified admissions.*—The number of certified patients admitted in the course of the year is a fair index of the occurrence of the severe types of mental illness. There appear to have been some increase in both male and female certified admissions in 1940, the male figures being the highest for seven years, the female for six. Probably the female number is about the average having regard to the number of patients discharged in September, 1939, and since re-certified. The male figure is higher and represents some increase that cannot be explained in this way, but taken in conjunction with the male voluntary patients it is not outstandingly high.

The table shows that contrary to expectation there has been no striking increase in the cases of mental diseases since the outbreak of the war, as reflected by the annual admission rate. The most significant finding in the table is the considerable reduction in the number of female and especially voluntary female patients admitted in the first year of the war. There was a reduction in male voluntary patients, but this was balanced by an increase in the number of certified males.

It is impossible to ascribe the fall in female admissions to any one cause. The greater opportunities for employment, even for individuals with little initiative or self-confidence, in well-paid work among large numbers of other persons, and the sense that that work has an ethical as well as a practical value must be of great importance. The idle and complaining member of the household gets little sympathy at home, and in the mental hospital fewer visits and less extra comforts sent in. Perhaps these altered conditions influence what has been called the malingering type of voluntary patient.

A more ordered life with prospects of settled employment removes a common cause of insecurity in younger women. Uniforms and badges have a certain glamour and endow the wearer with a sense of importance and recognition of personal worth or even indispensability previously not accorded. More than one former female patient is to-day in the A.R.P. services.

There is also more to do in the home. Ex-patients report how glad they are to look after children whose parents are working, to knit or to help after air-raids in their district. This gives them a common link with others after, perhaps, years of being misunderstood and excluded. Family and intimate social life have increased since entertainments, shopping and travelling are restricted. The necessity of extending hospitality to neighbours, friends and the homeless seems to have brought reality closer to the shut-in mind. The herd instinct has been stimulated by mutual misfortune and common danger. As one hypochondriacal woman said: "You can't think of yourself when everyone is going through so much."

Better wages and less unemployment among men allow more home comforts and a better standard of living in working-class homes. It is possible that for this reason in a number of households mental patients are being looked after rather than separated from the family.

Another surprising finding is the small number of temporary patients admitted during the year, suggesting that exhaustion and the various chances of the war are of no great account in the causation of psychic confusion. The stress and strain of the war, the fear of bombs, lack of sleep and the like are frequently cited by relatives as responsible for a patient's break-down. Examination of the male and female patients admitted during the first year of the war has failed to show that these were the only or the most important factors in many cases. During the recent heavy night raids no patients showed signs of panic, and certain cases, supposed to have suffered from air-raid terror, passed the nights without signs of fear.

It is interesting to note that the greatly altered conditions outside the Mental Hospital have not affected the recovery rate unfavourably. In 1940 118 patients were discharged "Recovered" apart from others "Relieved" and "Not Improved." This figure is 29 less than the highest, and 25 more than the lowest total for ten years, and the ratio of patients recovered to cases admitted, 33 per cent., is above the average. Since most patients spend a month at home on trial before obtaining the final discharge, these figures indicate that the incentive to remain at home and be useful is at least as great as in normal years.

*The chronic patients.*—The chronic population of the Mental Hospital accept the sirens and air-raid disturbances with indifference. Their withdrawal from society is complete enough to allow them to regard these interruptions as no more than a further folly perpetrated by the world to which they once belonged. Gunfire and sirens have less reality than a rather annoying film on the cinema screen has for the sane. However, many interesting reactions can be observed. These reactions are not wholly foolish: for example, the noisy and interfering patient finds a new joy in mimicking the sirens with startling perfection. Delusions of guilt are sometimes extended to include imagined offences against the black-out regulations or the giving away of secrets to the enemy. Perhaps the most unanimous reaction in the chronic wards is an angry disapproval of the warning bells which break the slumber that gun-fire fails to disturb.

### CONCLUSIONS.

1. During the first year of war there has been no increase in the number of patients admitted to the Mental Hospital. This suggests that the immediate psychogenic effect of the war has been negligible. Prolonged warfare may produce remote effects not yet observed.

2. Cases of acute confusion have been fewer than usual, and therefore do not depend to any extent on the stresses of war.

3. There has been a striking reduction in mental illness among female patients. The war appears to have supplied certain kinds of women with opportunities important for their mental health.

4. A year of war has shown no adverse effect on the annual recovery rate.

---

Having advanced these conclusions it may be interesting to consider briefly the experiences of others. Direct comparison is not possible for up to the present no papers have yet been published from another mental hospital. The majority of municipal mental hospitals have undergone some re-arrangement since the war as part of the emergency hospital scheme, some have been evacuated and some have been bombed, so that a statistical comparison between the years of peace and war must in most cases be difficult. It is to be hoped, but not perhaps expected, that the Bristol Mental Hospital will still be able to provide uncontaminated ground for study in the subsequent years of war.

Pegge,[1] Wilson[2] *et al.* found that there were notably few acute psychiatric casualties in their experience of severely bombed areas in London up to the end of September, and that casualties were rare amongst local defence workers and firemen who were probably much exposed to danger. In Coventry the normal weekly attendances at psychological out-patient clinics has dropped during the war and has shown no tendency to rise since the heavy raids on that city. Making allowances for the voluntary evacuation of neurotic and timid individuals, there can have been no appreciable immediate increase in psychiatric cases in Coventry (Massey).[3]

The only member of the civil defence services admitted to the Bristol Mental Hospital in 1940 was an A.R.P. warden suffering from the direct effects of blast. This case and problems allied to it are dealt with in another paper. Our experience in Bristol is probably similar to that of corresponding cities.

In 1920 Norgate[4] expressed his views on "the effects of the war on the mental conditions of the citizens of Bristol." It is unfortunate that his case material is not identical with that of the Mental Hospital. Early in 1915 the Mental Hospital, Fishponds, was evacuated to make room for military casualties, and many of the patients were dispersed throughout the country. A proportion of the more severe cases of mental disorder occurring subsequently were not treated in Bristol. Norgate's experience was confined to the inmates of Stapleton and Eastville Institution, where, in addition to cases of mental illness, mental defectives, alcoholics. "casuals," and the like were cared for. He believed that the war had operated unfavourably in mental health, but he had obviously no peace-time statistics for comparison.

His paper suggests that anti-social behaviour and alcoholism, probably amongst the defective types, were prevalent, but he found that by the end of the war alcoholism had become rarer, a finding he attributed to the increased cost and diminished potency

C

of the liquor then available. That depressive illnesses cannot have been greatly influenced by the war is suggested by the fall in the number of annual suicides in the years 1914 to 1918. Almost twice as many individuals committed suicide in 1913 as in 1918. Norgate has not failed to remark that the last war brought opportunities for occupation and employment to unhappy, dissatisfied women and ageing men, one benefit at least that has appeared again out of the evils of 1940.

### BIBLIOGRAPHY.

[1] Pegge, G., *Brit. Med. Jour.*, 1940, ii. 552.

[2] Report of Meeting at Tavistock Clinic, *Brit. Med. Jour.*, 1940, ii. 756.

[3] Massey, A., *Brit. Med. Jour.*, 1941, 1. 82.

[4] Norgate, R. H., *Bristol Med.-Chir. Jour.*, 1920, vol. xxxvii. 95.

